# Primary care-based multifaceted, interdisciplinary medical educational intervention for patients with systolic heart failure: lessons learned from a cluster randomised controlled trial

**DOI:** 10.1186/1745-6215-10-68

**Published:** 2009-08-13

**Authors:** Frank Peters-Klimm, Stephen Campbell, Thomas Müller-Tasch, Dieter Schellberg, Goetz Gelbrich, Wolfgang Herzog, Joachim Szecsenyi

**Affiliations:** 1 Department of General Practice and Health Services Research, University Hospital Heidelberg, Heidelberg, Germany; 2 Department of Psychosomatic and General Internal Medicine, University Hospital Heidelberg, Heidelberg, Germany; 3 National Primary Care Research and Development Centre, University of Manchester, Manchester, UK; 4 Coordination Center for Clinical Trials, University of Leipzig, Leipzig, Germany

## Abstract

**Background:**

Chronic (systolic) heart failure (CHF) is a common and disabling condition. Adherence to evidence-based guidelines in primary care has been shown to improve health outcomes. The aim was to explore the impact of a multidisciplinary educational intervention for general practitioners (GPs) (Train the trainer = TTT) on patient and performance outcomes.

**Methods:**

This paper presents the key findings from the trial and discusses the lessons learned during the implementation of the TTT trial. Primary care practices were randomly assigned to the TTT intervention or to the control group. 37 GPs (18 TTT, 19 control) were randomised and 168 patients diagnosed with ascertained CHF (91 TTT, 77 control) were enrolled. GPs in the intervention group attended four meetings addressing clinical practice guidelines and pharmacotherapy feedback. The primary outcome was patient self-reported quality of life at seven months, using the SF-36 Physical Functioning scale. Secondary outcomes included other SF-36 scales, the Kansas City Cardiomyopathy Questionnaire (KCCQ), total mortality, heart failure hospital admissions, prescribing, depressive disorders (PHQ-9), behavioural change (European Heart Failure Self-Care Behaviour Scale), patient-perceived quality of care (EUROPEP) and improvement of heart failure using NT-proBNP-levels. Because recruitment targets were not achieved an exploratory analysis was conducted.

**Results:**

There was high baseline achievement in both groups for many outcomes. At seven months, there were no significant mean difference between groups for the primary outcome measure (-3.3, 95%CI -9.7 to 3.1, p = 0.30). The only difference in secondary outcomes related to the prescribing of aldosterone antagonists by GPs in the intervention group, with significant between group differences at follow-up (42 vs. 24%, adjusted OR = 4.0, 95%CI 1.2–13; p = 0.02).

**Conclusion:**

The intervention did not change the primary outcome or most secondary outcomes. Recruitment targets were not achieved and the under-recruitment of practices and patients alongside a selection bias of participating GPs, prohibit definite conclusions, but the CI indicates a non-effectiveness of the intervention in this sample. We describe the lessons learned from conducting the trial for the future planning and conduct of confirmatory trials in primary care.

**Trial registration:**

ISRCTN08601529.

## Background

Chronic (systolic) heart failure (CHF) is a "common, disabling, deadly, and costly disease" often resulting in hospital admissions with a prevalence rate of approximately 1% in Germany[1,2]. New treatments and care strategies tend to focus on the prevention of hospital admissions and improving prognosis. The adherence of physicians to clinical practice guidelines (CPG)[3] and patients to treatment regimens [4], have been found to be predictors of better patient outcomes[5]. However, there are barriers[6] that must be overcome in order to ensure adequate communication between physicians and patients[7] and in the delivery of evidence-based care [8-10]. Furthermore, deteriorating CHF is associated with decreasing quality of life (QoL)[11,12]. Studies suggest that QoL is predictive of the course of CHF, independent of established somatic predictors of prognosis (such as the left ventricular ejection fraction)[12,13]. Increasing evidence shows that psychological comorbidities also determine QoL in patients with CHF[14].

In recent years, strategies designed to translate this evidence in to practice have consisted of changes in the organisation, delivery and specialisation of care by trained nurses[15], pharmacists[16], and call-centres providing home-based or telephone support or telemedicine [17-19]. These studies have primarily been conducted in a post-discharge setting and addressed the importance of adherence, recognising and acting upon deteriorating symptoms and optimising established pharmacological treatments.

The majority of CHF patients have contact with General Practitioners. However, the evidence for the effectiveness and efficiency of general practice based clinical practice guidelines is limited [20-22]. Combinations of interventions seem most promising[23], along with evidence-based educational strategies for physicians[24,25]. However, interventions that are designed to change GP behaviour must be realistic in terms of the effects of educational strategies on GP knowledge during their careers[26].

The purpose of this study was to develop an educational model for GPs for the management of CHF and to evaluate it as randomised controlled trial. The original aim was to conduct a confirmatory trial. However, under-recruitment of participants meant that we conducted an exploratory trial of a complex intervention combining educational sessions with medication feedback. This paper reports the main findings from the trial and focuses upon the lessons learned from its planning and conduct.

## Methods

### Participants – Recruitment and Assignment

In a single mail-out, we invited GPs from the region of Northern Baden, Germany, to participate in the study. GPs were eligible for participation if they were certified as a GP and practised as a SHI (statutory health insurance)-affiliated physician. GPs were excluded if they provided unconventional therapies or provided specialist services (e.g. HIV-therapy).

GPs were expected to recruit an average of 7–8 patients in to the study. GPs received a screening algorithm for case finding using electronic medical records. Eligible patients were adults (aged over 40 years) with objective left- or biventricular heart failure, NYHA functional class II-IV, with ascertained ejection fraction of 40% or less (e.g. by echocardiography), with stable symptoms at the time of inclusion, and diagnosis of a chronic, irreversible CHF at least 3 months prior to inclusion. We excluded patients with primary valvular heart diseases and relevant hemodynamic effects, hypertrophic obstructive/restrictive cardiomyopathy (HOCM/RCM), and people with a concomitant terminal illness, dementia or severe psychological illness. We obtained informed consent from all participants.

After recruitment of practices and patients, stratified randomisation of practices was carried out by an external third party using the "single-coin method" based on a computer generated list. The third party stratified practices according to the number of participating patients per practice. Intervention allocations were concealed from particpating GPs and the intervention team by the third party until shortly before the intervention started in October 2005.

### Intervention

**Figure 1:**
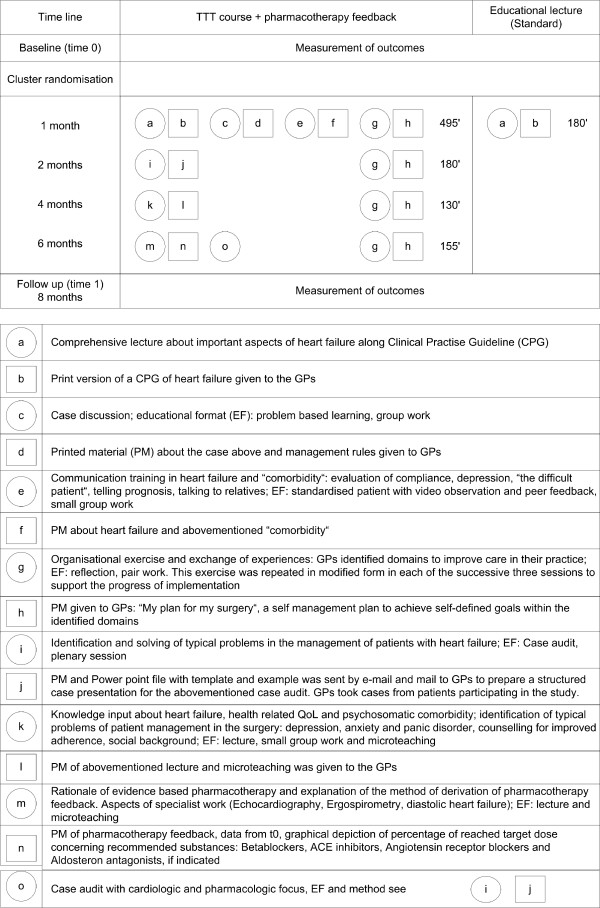
**Graphical depiction of the (i) timeline, (ii) composition and duration (' = minutes), (iii) content and (iiii) educational format (EF) of the educational interventions; PM = printed material**. Objects are represented by squares (to reflect their fixed nature, e.g. handout of printed material) and activities by circles (to reflect their flexibility, e.g. group work). Different components are labelled using different letters.

We conducted a focus group with 13 GPs who were not actively participating in the TTT study. The content of the educational sessions was based on the discussions of this focus group. Both educational interventions were based on predefined learning targets relevant to heart failure including psychosomatic aspects (comorbid depression and anxiety disorders, quality of life, and compliance). We focused on the optimisation of evidence-based pharmacotherapy and the detection of depressive comorbid disorders in patients with CHF. The intervention team consisted of one GP, one cardiologist and one specialist in psychosomatic medicine. The new intervention group (Train the trainer = TTT) participated in a multidisciplinary, andragogic (e.g. inputs of theoretical knowledge with group work in "sandwich technique"), and didactic (e.g. problem-based learning) training course including elements of specific knowledge (e.g. CPG content), communication skills (standardised patients with video observation and peer feedback), detection and management of comorbid mental disorders (diagnostic properties of screening tools) as well as organisation of the practice in order to implement newly acquired knowledge ("my plan for my practice"). For the same reason, structured case-discussions were held on real patients. The interventions are depicted in Figure 1, as recently recommended[27]. Additionally, GPs in the TTT group received medication feedback for individual patients participating in the study (data from baseline documentation of patients). The existing percentage of patients receiving the target dose of ACE-inhibitors (or angiotensin-II receptor antagonists) and beta-blockers was calculated using current guidelines as shown previously and printed out as a graphical depiction for each patient[28].

GPs from the control group received a single three-hour lecture by a senior cardiologist with extensive didactic expertise based on the aforementioned predefined learning targets.

### Masking

The nature of the intervention entailed that participating physicians and individuals of the intervention team were unblinded, while patients were not informed about group assignment. No interim analyses were conducted during the observation period, with the exception of data collection on heart failure medication for pharmacotherapy feedback. Neither the study statisticians nor the data monitoring committee saw unblinded data or had any form of contact with study participants.

### Objectives

We investigated whether the intervention would improve health-related quality of life (QoL), reduce hospital admissions or mortality, improve self care behaviour, improve satisfaction with provided care and improve disease course in patients with CHF. Compared with the control group, we hypothesised that the new intervention would result in an improvement in QoL (SF-36, Physical Functioning scale) of at least 6.6 points.

### Outcomes

The primary outcome was self-assessed quality of life at follow-up at seven months, i.e. Subscale 1 (Physical Functioning) of the German version of the SF-36[29,30] (a generic, multidimensional instrument). Secondary outcomes included further SF-36 subscales, the German version of the Kansas City Cardiomyopathy Questionnaire (KCCQ)[31] (a disease-specific instrument) (KCCQ-**os**). A mean five-point change in the scales of the SF-36[30,32] and in the **o**verall **s**ummary score (KCCQ-os)[33] represents a clinically significant difference. Further pre-specified secondary outcomes were total mortality or heart failure hospital admissions, depressive disorders measured using the PHQ-9[34,35], the European Heart Failure Self-Care Behaviour Scale (EHFScBS)[36], patient-perceived quality of care (EUROPEP)[37], and improvement of heart failure according to NT-proBNP-levels[38,39].

### Data collection

The trial was part of the Competence Network of Heart Failure and conducted as a clinical trial according to the principles of ICH/GCP (for example: a list of involved study personnel, screening of eligible patients, informed consent, registration and pseudonymisation of patients)[40]. GPs received an initiation visit by a study nurse, which included an introduction to the trial's investigator file. GPs collected and documented clinical data (history, current clinical status), laboratory results, ECG, detailed medication etc., discontinuation of the study by the patient, and mortality data on pre-specified case report forms according to the Basic Clinical Dataset (BCD) of the Competence Network of Heart Failure a nationwide interdisciplinary research project involving medical doctors and scientists from university and other clinics, research institutes, heart centers, medical practices as well as organisations, associations and industry [41]. This network aims to obtain an additional value for patients, clinicians and scientists by multidimensional connection of these numerous partners by conducting – amongst others – clinical trials). They also took blood samples for the determination of NT-proBNP at baseline and follow-up. NT-proBNP was measured using the Elecsys 2010 analyser from Roche Diagnostics, Germany. GPs also documented the number of practice visits by patients, referrals to a cardiologist, and hospital admissions.

### Sample size calculation

For the primary outcome, we took into account the expected small effect of the intervention and the natural deterioration of QoL, esp. in the scale physical functioning. Therefore, we assumed mean small decline of 3.3 points in the control group due to the natural disease trajectory and a small mean improvement under the intervention by 3.3 points, resulting in the expected net difference of 6.6 points in QoL in favour of the intervention. The standard deviation from cross-sectional analyses is reported to be 23[30]. Using the serial correlation from baseline to follow-up measurements of 0.6[30], we can calculate the anticipated variance (SD) of the difference of 423.2 (20.57). Therefore, in a two-sided sample calculation for 80% power at an α-level of 5%, 154 patients were needed per arm. To account for the clustered design, the literature indicated that intra-class correlation coefficients (ICC = GP variance/[GP variance + patient variance]) of health outcomes (like QoL) are generally lower than 0.05[42,43]. As the German primary care is characterised by small, office-based practices, we could expect that each participating GP would come from another practice. Our assumption was that the mean change of GP would deviate less than ± 5 points from the overall mean in 95% of all practices. This results in a SD (Variance) of GP of 2.5 (6.25) and an ICC of 0.015 to account for the variable responses of GPs and patients to the intervention. Thus, with k = number of GPs (60), n = sample-size from conventional planning (308), we obtain the total N = k*n*(1-ICC)/(k-n*ICC) = 329. We anticipated a drop out rate of 30%[2], as a result of which 329 cases would provide 70% power. Accordingly, 329*10/7 = 470 cases represented 100%. In order to obtain a total sample of 470 patients, we planned to include 60 practices with 7–8 patients per practice, assuming a practice prevalence of 1% similar to the estimated average prevalence in the German population[2,44].

### Statistical methods

In order to identify group differences in the baseline characteristics of GPs and patients, we used chi-square and t-tests. For continuous dependent variables (score at follow-up), comparisons between the intervention and control practices were assessed using linear mixed effects regression models (SAS 9.1 proc mixed) with the practice as a random effect nested in groups. These analyses accounted for the intraclass correlation within each practice attributable to clustering from randomisation according to practice. The analysis model included the fixed effect group (intervention vs. control), and the covariates score at baseline, age, sex, and NYHA functional class.

We analysed the effect of the intervention on binary outcomes using SAS proc glimmix and generalised linear mixed effects models with a logit link. We analysed the effect of the intervention on count-data outcomes using generalised linear mixed effects models with a poisson link, and accounting for overdispersion. Continuous outcomes are presented as adjusted mean differences and binary or count outcomes as adjusted odds-ratios (ORs) with 95% confidence intervals (CIs).

## Results

### Participant flow and follow-up

**Figure 2:**
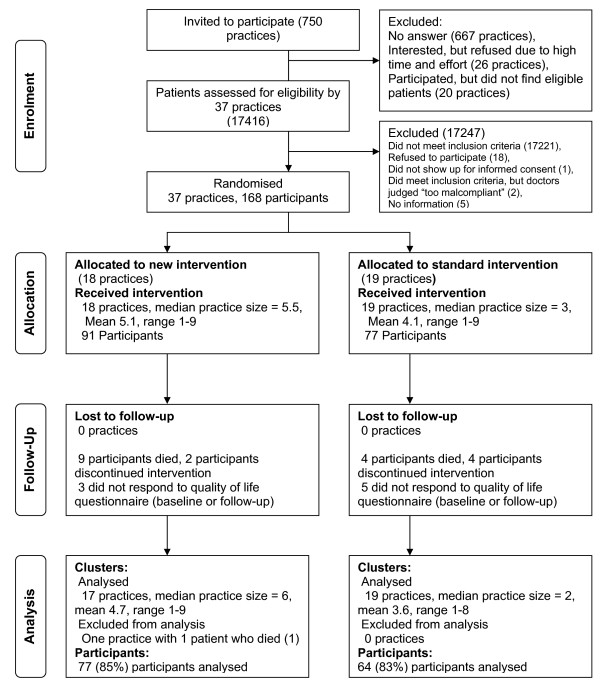
**Flow of clusters and participants through the trial**.

Figure 2 shows the flow of participating practices (=GPs) and patients through the trial. We approached a total of 750 GPs in a single mail-out in October 2004. 667 did not respond; 26 were interested, but finally refused to participate due to work load; and 20 failed to find eligible patients. 37 GPs ultimately participated. Between March and September 2005, these GPs screened 17416 patients for eligibility and ultimately recruited 168 eligible patients in 37 practices. Following patient recruitment, we randomised 18 GPs to the TTT group and 17 GPs to the Standard care group. Both groups displayed similar cluster sizes. Some recruiting GPs failed to recruit the intended number of eligible patients. At the participant level, 14 patients were ultimately lost in the TTT group and 13 in the Standard group (for details see Figure 2). Table 1 shows similar characteristics for GPs in the TTT and Standard group. Table 2 shows similar baseline characteristics for participants in the TTT and Standard group [45].

**Table 1:** Baseline characteristics of all 37 participating general practices from Baden-Wuerttemberg.

	Intervention group (n = 18)	Control group (n = 19)
Practice factors at baseline		

Female GPs	3 (17)	4 (21)

Age of GPs in years (SD)	50 (9.4)	50 (5.9)

Certification of GPs since years (SD)	16 (11.4)	15 (7.2)

No. of GPs (whole time equivalent)		

Single	9 (50)	11 (57.9)

Two	8 (44.4)	5 (26.3)

More than two	1 (5.6)	3 (25.8)

Location		

Rural	13 (72.2)	9 (47.4)

Suburban	2 (11.1)	4 (21.1)

Urban	3 (16.7)	6 (31.6)

List size (patients per quarter)		

0–999	6 (33.3)	3 (15.8)

1000–1499	5 (27.8)	8 (42.1)

>1499	7 (38.9)	7 (36.8)

Participation in disease management programmes or quality circles	17/18 (94.4/100)	19/18 (100/94.7)

Values represent number (percentages) of practices unless stated otherwise

**Table 2:** Baseline comparison of intervention and control group patients (n = 168).

	Intervention group (n = 91)	Control group (n = 77)
Male sex	63 (69.2)	53 (68.8)

Mean (SD) age (years)	68.4 (10.6)	69 (9.5)

Living alone	27 (29.4)	22 (28.6)

No information	3 (3.7)	11 (14.3)

Social class*:		
Mean (SD) score	8.9 (3.8)	8.8 (4.3)
lower, middle, upper class	46 (52.3),	34 (54),
	36 (40.9),	21 (33.3),
	6 (6.8) (n = 88)	8 (12.7) (n = 63)

NYHA-functional class (according to GP)		

II	44 (48.4)	41 (53.3)

III	46 (50.6)	33 (42.9)

IV	1 (1)	3 (3.9)

Mean (SD) LVEF	32.5 (7.1) (n = 79)	34.4 (6.5) (n = 64)

Main cause of CHF		

CHD	43 (47.3)	31 (40.3)

Cardiomyopathy	29 (31.9)	21 (27.3)

Hypertension	6 (6.6)	15 (19.5)

Not clear	13 (14.3)	10 (13)

Mean (SD) duration (years) of CHF	5.6 (4.9)	5.8 (5.6)

Localisation of CHF		

Left	55 (60.4)	50 (64.9)

Left and right	31 (34.1)	24 (31.2)

Unknown	5 (5.5)	3 (3.9)

Cardiovascular interventions		

PTCA/Stent (any)	37 (40.7)	19 (24.7)

Bypass (any)	23 (25.3)	20 (26)

Pacemaker (any)	18 (19.8)	13 (16.9)

ICD	19 (20.9)	11 (14.3)

Reanimation/Defibrillation	7 (7.7)	7 (9.1)

Medical conditions		

Atrial fibrillation	21 (23.1)	12 (15.6)

PAD	15 (16.5)	14 (18.2)

Cerebrovascular disease	18 (19.8)	14 (18.2)

COPD	18 (19.8)	15 (19.5)

Depression	22 (24.2)	17 (22.1)

Creatinine-Clearance: Mean (SD) GFR (ml/min)**	74.1 (31.7)	66.5 (27.4)

Cardiovascular risk factors		

Diabetes mellitus	32 (35.2)	29 (37.7)

Hypertension	68 (74.7)	60 (77.9)

Dyslipidemia	68 (74.7)	60 (77.9)

Hyperuricemia	44 (48.4)	33 (42.9)

(Ex-)smoker (since at least 6 months)	34/17 (37.4/18.7)	34/9 (44.2/11.7)

Drugs at baseline included:		

ACE inhibitor/A2RA	83 (91.2)	68 (88.3)

β-blocker	71 (78.0)	62 (80.5)

ACE inhibitor/A2RA and β-blocker	65 (71.4)	57 (74.3)

Spirononolactone/Eplerenone	29 (31.9)	19 (24.7)

Loop diuretics	55 (60.4)	47 (61)

Cardiac glycosides	32 (35.2)	32 (41.6)

Antidepressants	7 (7.8)	5 (6.5)

Soporifics/hypnotics	7 (7.7)	3 (3.9)

Values represent number (percentages) of patients unless stated otherwise.

*Social Class according to modified German Winkler-index[45] (lower class: 3–7; middle class: 8–14; upper class: 15–21); NYHA, New York Heart Association; LVEF, Left ventricular ejection fraction; CHF, Chronic (systolic) heart failure; CHD, Coronary heart disease; PTCA, Percutaneous Transluminal Coronary Angioplasty; ICD, implantable cardioverter defibrillator; PAD, Peripheral arterial disease; COPD, Chronic obstructive pulmonary disease; ** Estimation of the GFR according to the formula of Cockroft and Gault; ACE = angiotensin converting enzyme; A2RA = angiotensin-2 receptor antagonist;

### Interventions

Only few participating GPs from TTT sporadically missed one of the educational sessions, all of which were held at the University of Heidelberg. The IG (n = 18) was divided into two subgroups: TTT subgroups (n = 10/n = 8) came to the educational meetings in October (10/8) and November 2005 (8/8), and – after new group formation – in January (7/9) and March (9/8) 2006. The Standard group (n = 19) attended a lecture in October 2005.

### Psychosocial outcomes

Table 3 shows all patient-reported outcomes at baseline and at seven-month follow-up. These were the scores for *Quality of life *(SF36- and KCCQ), *Behaviour change *(EHFScBs), *Patient satisfaction *(EUROPEP), and *Comorbid Depression (PHQ-9)*.

**Table 3:** Mean generic quality of life (SF-36), disease-specific quality of life (KCCQ), European heart failure self-care behaviour scale (EHFScBs), patient satisfaction (EUROPEP) and depression status (PHQ-9-D) scores for groups at baseline and seven-month follow-up

		**Intervention group**		**Control group**		**No**	**ICC**	
		
		Score (SD)	No	Score (SD)	No			Adjusted mean difference** (95% CI); p***-value
SF-36 scales*								

Physical functioning	Baseline	52.2 (27.7)	77	45.5 (27.8)	64			
	
	Seven months	44.9 (28.9)	77	43.6 (26.3)	64	141	0	-3.3 (-9.7 to 3.1); 0.30

Role functioning, physical	Baseline	42.5 (44.4)	67	34.3 (41.3)	54			
	
	Seven months	31.3 (41.5)	75	37.9 (43.5)	60	119	0.12	-5.9 (-19.7 to 7.8); 0.30

Bodily pain	Baseline	63.2 (27.9)	76	60.5 (29.5)	64			
	
	Seven months	58.5 (29.9)	76	53.8 (29.2)	64	139	0.000	3.6 (-5.6 to 12.7); 0.43

General health perceptions	Baseline	48.3 (17.8)	77	44.2 (19.2)	63			
	
	Seven months	44.9 (18.6)	74	44.3 (18.2)	64	137	0.000	-2.0 (-7.5 to 3.6); 0.47

Vitality	Baseline	43.9 (21.3)	75	43.9 (22.5)	63			
	
	Seven months	39.0 (21.3)	77	43.1 (20.4)	63	137	0.070	-4.1 (-11.1 to 2.7); 0.22

Social functioning	Baseline	75.3 (23.2)	77	67.3 (27.4)	63			
	
	Seven months	64.0 (31.4)	77	64.8 (28.9)	64	140	0.10	-5.6 (-16.8 to 5.6); 0.31

Role functioning, emotional	Baseline	65.9 (45.2)	68	66.0 (46.0)	54			
	
	Seven months	56.3 (47.0)	71	60.3 (46.5)	58	117	0.000	-2.6 (-18.1 to 12.9); 0.73

Mental health	Baseline	62.3 (20.2)	75	66.0 (20.3)	62			
	
	Seven months	58.9 (21.7)	77	63.8 (20.0)	63	136	0.002	-1.8 (-8.0 to 4.4); 0.56

KCCQ* overall summary								

	Baseline	66.7 (20.9)	78	63.6 (20.2)	60			-2.4 (-9.3 to 4.4); 0.47
	
	Seven months	64.0 (23.7)	78	65.3 (20.9)	64			

EHFScBs****								

	Baseline	24.4 (7.8)	90	23.8 (7.5)	70			1.3 (-0.9 to 3.5); 0.24
	
	Seven months	24.1 (7.9)	76	23.6 (6.8)	64			

EUROPEP*****								

	Baseline	105.8 (9.3)	90	106.2 (8.7)	69			-0.5 (-4.1 to 3.2); 0.80
	
	Seven months	104.8 (10.4)	77	104.5 (10.8)	63			

PHQ-9******								

sum score	Baseline	7.4 (5.6)	91	7.4 (5.8)	76			
	
	Seven months	8.6 (5.9)	77	7.4 (5.4)	63			0.8 (-0.7 to 2.3); 0.30

								adjusted odds ratio******* (95% CI); p***-value

No. (%) of Major Depressive Syndromes	Baseline	12 (15.6)	77	7 (11.1)	63			
	
	Seven months	14 (18.2)	77	8 (12.7)	63			1.5 (0.5 to 4.6); 0.48

*High scores imply better health.

**Based on analysis of covariance comparing results at seven-month follow-up.

***Adjusted for baseline score, ICC, New York Heart Association class, gender and age.

****Scores range from 12–60; low scores imply better self-care behaviour.

*****Scores range from 23–115; higher scores imply higher patient satisfaction regarding provided care.

******For details see methods section

*******According to generalised linear mixed effect models with a logit link using SAS proc glimmix to compare the number of outcomes between groups

### Somatic and performance outcomes

#### Mortality or hospital admissions

Mortality data were available for all participants. More deaths occurred in the TTT group than in the Standard group (9 vs. 4) (Table 4), although this difference was not statistically significant (adjusted odds ratio = 2.0, 95% confidence interval 0.6 to 7.1; P = 0.27). A total of 31 heart failure admissions occurred in the TTT group and 34 in the Standard group (adjusted odds ratio = 0.8, 95% confidence interval 0.3 to 2.1; P = 0.63). The combined end point (mortality or heart failure admission) resulted in 29 cases in the TTT group and 18 cases in the Standard group. The Poisson model indicated that this difference between groups was not significant (adjusted odds ratio = 1.4, 95% confidence interval 0.7 to 2.9; P = 0.35).

**Table 4:** Heart failure hospital admissions and total mortality during seven-month trial follow-up, selected prescription rates for patients at seven-month trial follow-up, mean NT-proBNP-values at baseline and seven-month follow-up and primary care activity data during seven-month trial follow-up

**Outcome**	**Time**	**Intervention group**		**Control group**		
		
			**No**		**No**	
						Adjusted odds ratio* (95% CI); p**-value

**Death (any cause)**	Seven months	9	91	4	77	2.0 (0.6 to 7.1); 0.27

**Patients admitted **to hospital due to **heart failure, no. (%)**	Seven months	23 (27.1)	85	16 (23.5)	68	1.2 (0.5 to 2.6); 0.67

**No. of heart failure hospital admissions**	Seven months	31	85	34	68	0.8 (0.3 to 2.1); 0.63

**Heart failure hospital admission or death**	Seven months	29	89	18	70	1.4 (0.7 to 2.9); 0.35

**No. (%) of drugs**			85		68	adjusted odds ratio*** (95% CI); p**-value

ACE inhibitor or A2RA	Seven months	78 (91.8)		61 (89.7)		0.9 (0.2 to 3.4); 0.87

β-blocker	Seven months	68 (80)		57 (83.8)		0.7 (0.2 to 2.7); 0.58

ACE inhibitor/A2RA and β-blocker	Seven months	65 (76.5)		53 (77.9)		0.8 (0.2 to 2.9); 0.78

Spirononolactone/Eplerenone	Seven months	36 (42.4)		16 (23.5)		**4.0 (1.2 to 13.0); 0.02**

Mean **NT-proBNP**- values (pg/ml) (SD)						Adjusted mean difference**** (95% CI); p**-value

Crude	Baseline	2462.5 (2821.5)	87	2732.2 (5793.5)	69	
						
Transformed*****		4.3 (1.0)		4.1 (1.1)		

Crude	Seven months	2031.6 (3575.4)	71	1411.7 (2218.1)	57	0.17 (-0.04 to 0.39); 0.11
						
Transformed*****		3.9 (1.1)		3.6 (1.1)		

**Mean practice attendances **(SD)	Seven months	24.0 (16.0)	80	21.6 (15.3)	67	3.9 (-2.9 to 10.7); 0.25

**Mean referrals to cardiologist **(SD)	Seven months	2.2 (2.5)	85	2.2 (1.9)	68	-0.03 (-0.8 to 0.7); 0.93

*Poisson regression model comparing number of outcomes between groups

**Adjusted for recruitment site (cluster), baseline value, New York Heart Association class, age and gender

***According to generalised linear mixed effect models with a logit link using SAS proc glimmix to compare the number of outcomes between groups

****Based on analysis of covariance comparing results at seven–month follow-up

***** Due to a skewed distribution of NT-proBNP, logarithmic transformation was performed. Approximation to a normal density was achieved using the formula *t *= 2*log_10_(*B*+10)-2, where B denotes the raw NT-proBNP value[39]

#### Primary care activity data

Practice attendances by patients and referral rates are shown in Table 4. There were no statistically significant differences between the groups. Prescription rates for evidence-based pharmacotherapy at follow-up were high, e.g. for the combination of an ACE inhibitor or an angiotensin-2 receptor antagonist with a β-blocker we found a rate of 76.5% for the IG and 77.9% for the CG, although this group difference adjusted for baseline rates (adjusted odds ratio 0.8, 95% confidence interval 0.2 to 2.9; P = 0.78) was not significant. With a prescription rate of 42.4% for aldosterone antagonists at seven-month follow-up, TTT showed a significantly greater increase than Standard with a prescription rate of 23.5% (adjusted odds ratio 4.0, 95% confidence interval 1.2 to 13.0; P = 0.02).

#### NT-proBNP

Mean NT-proBNP plasma levels (SD) were 2462.5 pg/ml (2821.5) in TTT (n = 87) and 2732.2 pg/ml (5793.5) in Standard (n = 69) (Table 4). Mean levels decreased in both groups. There was a non-significant difference between the groups (adjusted mean difference for the transformed levels = 0.17 points, 95% confidence interval 0.04 to 0.39; P = 0.11).

## Discussion

### Summary of main findings

The low power of the trial due to under-recruitment does not allow definitive conclusions regarding the effectiveness of the intervention in terms of the primary outcome (generic QoL, i.e. scale physical functioning of SF-36), but failed to show effectiveness in a seven-month follow-up. The intervention combined multifaceted, interdisciplinary educational sessions with pharmacotherapy feedback for GPs and was compared to a control group that received only a standard lecture. In terms of further patient-reported outcomes, we found impaired QoL, but good self-care and a generally high level of satisfaction with care in both groups, with no significant differences or changes. The intervention led to a significantly higher increase in the prescription rate of aldosterone antagonists and seemed to lead to a concomitant increase in primary care activity (by increase of practice attendances).

The following discussion is structured along the descriptive and exploratory character of the study and includes the lessons we have learned from planning and conducting the trial.

### Comparison with existing literature

We compared QoL, self-care, patient satisfaction with care and performance measured by guideline adherence. Our study confirms that patients with CHF report impaired QoL[11]. Trials with a positive impact on the QoL in heart failure patients, typically use complex and multidisciplinary interventions at the *patient *level (heart failure programmes) in a post-discharge setting[46,47]. These patients were by definition unstable as they were hospitalised due to heart failure and more symptomatic ("ill") with a lower QoL at enrolment, while not necessarily more "diseased", as indicated by the past medical history and substantial comorbidities[46,47]. Patients in our trial were enrolled in primary care and had stable CHF at enrolment.

Although reporting comorbidity and impaired QoL scores, our patient sample revealed a higher level of self-care than previously reported[36]: Mean sum scores of around 24 (SD: 6.8–7.9) were better than in the sample from the validation study in which 442 patients from two centres in Sweden, three in the Netherlands and one in Italy were included[36]. Mean sum scores were 33.3 (7.8) vs. 29.6 (9.0) for subgroups extra care vs. standard care (defined by extra patient education about heart failure). Data on self-care from a trial testing community pharmacists showed similar high ("bad") baseline scores: Mean (SD) scores were 31.1 (8.7) vs. 30.6 (9.1) at baseline. Self-care improved in both groups at three and six month's follow-up (26.1 vs. 26.6 and 26.6 vs. 28.3, respectively). An intensive heart failure programme in a physician-nurse directed heart failure clinic had better, similar baseline scores to our sample (23.6 vs. 25.5 for intervention and control groups). The intervention improved self-care in a 3 months follow up, while scores at 12 months were similar as at baseline again. Still, there were significant between group differences because of worsening self-care scores in the control group (30.2 at 12 months). This worsening self-care was not found in our (active) control group (at 7 months).

The high overall satisfaction with care scores in our sample are similar to those found in the German sample in a European comparative patient survey in general practice using the EUROPEP instrument[37].

Comparable educational interventions in the CME setting have proven their efficacy in other clinical fields[24,25]; outcomes are typically knowledge or performance based rather than using patient-related outcomes: For CHF, educational programmes for GP peer review groups have not been shown to be beneficial for performance as assessed by prescription behaviour[48]. Compared with the literature (from 2002), GPs in our study (2005) showed high guideline adherence with regard to the prescription of evidence-based pharmacotherapy[8]. Even more recent studies suggest potential for improvement with regard to evidence-based pharmacotherapy [48-50], although the samples in these studies are of minor validity due to a lack of ascertained left ventricular systolic dysfunction (LVSD) and therefore not directly comparable. LVSD remains the crucial indication for prescribing evidence-based pharmacotherapy, preferably towards target doses[3,9,51]. Therefore, a rationale for the TTT-intervention was that patients with LVSD and a suspected low level of care could benefit from optimised treatment[3,51]. However, there is still a lack of evidence for the effectiveness of (both non-pharmacological and pharmacological) treatment of patients with "undefined" (e.g. diastolic) heart failure ("elderly female patient in general practice")[52].

In our patient sample, prescription rates of ACE-inhibitors (or A2RA) and beta-blockers were "already" very high at baseline, allowing primarily up-titration towards target doses[28]. This effect of TTT could be shown in a separate, more detailed analysis on guideline adherence[53]. Generally, interventions to improve guideline adherence to the prescription and target dosing of ACE-inhibitors (a hospital-based intervention) [54] or beta-blockers with mixed results [55,56] are more successful if they succeed in professional engagement[55]. The latter was the case in our tailored intervention.

### Strengths and limitations of the study

#### Internal Validity

The processes of recruiting practices and patients and concealed randomisation, ensured equivalent groups of both GPs and patients at baseline and there was a good follow-up rate of patients (84% for the primary outcome). However, it is not possible to blind GPs to treatment group in such studies, which may have biased the activity of GPs as well as patient responses to questionnaires. Originally, the trial included a follow-up immediately before the intervention to detect any observer bias (the Hawthorne effect); we had to amend this design, however, on account of feasibility and work overload for the documenting GPs.

The insufficient recruitment of participating GPs and patients with left ventricular systolic dysfunction (LVSD) meant that we had to turn the trial, and its research question concerning the primary endpoint (QoL), into an exploratory trial.

#### External validity

We were not able to perform a non-responder analysis, but we have some indication that our sample of GPs was highly selective (Table 1): While the participating GPs were representative in terms of mean age and the number of GPs in one practice, they also had larger than average list sizes: the average list size in Germany is approximately 800 patients, and patients are free to change their doctor at any time. Almost all participating GPs adopted the German disease management programmes (average German participation rate approximately 53% in 2006) and also actively took part in peer review groups ("quality circles") (average German participation rate approximately 30% in 2006). Furthermore, a selection bias is supported by a high guideline-adherence at baseline regarding evidence-based pharmacotherapy (Table 2).

### Meaning of the study – lessons learned

Due to the low power of the study it is only possible to speculate on the possible reasons for the lack of effectiveness of the intervention on QoL. We identified the following possible reasons:

1. Testing against an unblinded and active control group hampers proving efficacy but this cannot be changed due to the nature of complex interventions and ethical considerations.

2. Given the indirect design of our study, with the intervention at the level of the GP and outcome measurement at the level of the patient, our follow-up may have been set too early: While all GPs received the TTT-course, our evaluation in month 6 showed that GPs had implemented approximately 30–40% of the self-determined implementation targets (Figure 1).

3. The intervention may have a (transient or permanent) negative effect because of up-titration[28,53] of β-blockers by GPs might have led to significant (transient) worsening of symptoms such as dyspnoea. Furthermore, increased activity of GPs in other contexts (including more scheduled attendances or serum level controls) could cause patients to perceive that they are more ill than was previously the case.

4. Our intervention was possibly carried out at a too late stage in the course of the disease as patients had a mean history of CHF of approximately six years. Improved patient outcomes have been shown in newly diagnosed patients or following a deterioration requiring hospital admission. Our patient sample shows a higher level of self-care behaviour than previously shown, suggesting that the patients in our study had already adapted their lifestyle to their diagnosis.

#### Lessons learned

In this trial, we encountered problems administering the study which we documented systematically along with possible solutions for how such similar problems could be dealt with in future studies (see Additional file 1, table S1).

Regarding the conceptual and planning phase of the trial, several factors hampered a tailoring the trial to general practice (see Additional file 1, table S1). The funding was too small for a clinical trial of this size and a longer intervention timeline, as recommend for complex interventions, could have had more impact[57].

In the operational phase we differentiated between the recruitment of investigators (GPs) and the recruitment of study participants. GPs were recruited from a single region of Germany (Northern Baden) and we encountered problems recruiting participants. Moreover, while we temporarily achieved the recruitment target of 60 GPs 26 GPs who had expressed an interest ultimately declined. Each GP was telephoned and asked for their reasons for not participating and the main reasons cited were the perceived workload and time commitment as well as the small financial incentive for participation. Extending the recruitment catchment area would have been a possible solution but was not feasible. In the UK, working with practice-based research networks (PBRNs) is considered a priority by national funding programmes, for example the Primary care research Network (PCRN)  or the Medical research council general practice research framework (MRCGPRF) . For more ambitious research questions, a German PBRN would offer a valuable solution with regard to the recruitment of practices. However, it would be problematical to generate a representative sample of practices in such a network.

Case finding was another important barrier to the sufficient recruitment of study patients, as 20 GPs failed to find any eligible patients despite substantial screening efforts. The different reasons are listed in table S1 (see Additional file 1).

The inclusion criterion LVSD was another barrier as was a lack of understanding of LVSD by GPs (see Additional file 1, table S1). Extending the catchment area of recruitment and funding for a mobile study echocardiography could have been potential solutions but were not feasible for logistical reasons.

Regarding the analysis, our actual ICCs revealed numbers close to 0, hence an ICC as small as 0.015 was a sufficiently conservative assumption. We additionally found a low mortality rate (7.7%), and considering the lower total drop-out rate, a sample size of 390 participants would have been needed.

## Conclusion

Due to considerable under-recruitment, the study was no longer satisfactorily powered to discover the pre-specified effect size. However, the observed data resulted in a confidence interval which definitely excludes a difference of 6.6 points in favour of the intervention group. Hence the study is conclusive in so far that the intended moderate treatment effect has been significantly refuted. Our study sample showed selection bias with high performing GPs with access to specialist care and patients that had adapted their self-care behaviour. Therefore, the study can be conceptualised as an exploratory trial[53]. The patient outcome results are valuable for future comparisons and could also be used for pooling in a meta-analysis.

The lessons learned are that measures implemented to increase internal validity (clinical trial design) were at the cost of study power and generalisability. No effective counter-measure was available at the time of the recruitment phase. For primary care-based clinical trials, our trial underlines the need for primary care research development and the establishment of research networks.

## Abbreviations

A2RA: Angiotensin-2 receptor antagonist; ACE: Angiotensin converting enzyme; CHD: Coronary heart disease; CHF: Chronic (systolic) heart failure; CI: Confidence interval; CME: Continuous medical education; COPD: Chronic obstructive pulmonary disease; CPG: Clinical practice guideline; EHFScBS: European Heart Failure Self-care Behaviour Scale; EUROPEP: **P**atients in **Euro**pe **e**valuate general **p**ractice care; GFR: Glomerular filtration rate; GP: General practitioner; ICC: Intra-class correlation coefficient; ICD: Implantable cardioverter defibrillator; KCCQ: Kansas City Cardiomyopathy Questionnaire; LVEF: Left ventricular ejection fraction; LVSD: Left ventricular systolic dysfunction; NT-proBNP: N-terminal Brain Natriuretic Peptide; NYHA: New York Heart Association; OR: Odds ratio; PAD: Peripheral arterial disease; PC: Primary Care; PHQ-9: Depression module of the **P**atient **H**ealth **Q**uestionnaire; PTCA: Percutaneous Transluminal Coronary Angioplasty; QoL: (Health-related) Quality of life; RCT: Randomised controlled trial; SF-36: MOS **36**-item **s**hort-**f**orm health survey; TTT: Train the trainer

## Competing interests

The authors declare that they have no competing interests.

## Authors' contributions

DS, TMT, WH, GG, JS, SC and FPK designed the study. DS and FPK analysed the results. All authors interpreted the results. FPK wrote the manuscript, and all authors contributed to writing revisions and approved the final manuscript. FPK is the guarantor.

## Supplementary Material

Additional file 1 **Table S1: Problems and possible solutions according to each phase of the clinical trial**. The table lists the problems encountered during the phases of the trial and its potential solutions Click here for file
