# Supplementary material for: Primary care-based multifaceted, interdisciplinary medical educational intervention for patients with systolic heart failure: lessons learned from a cluster randomised controlled trial
Source: Trials. 2009 Aug 13;10:68. doi: 10.1186/1745-6215-10-68 (PMC2736948; doi:10.1186/1745-6215-10-68)
Supplement: Additional file 1 — Table S1: Problems and possible solutions according to each phase of the clinical trial. The table lists the problems encountered during the phases of the trial and its potential solutions [file 1745-6215-10-68-S1.doc]

| **Phase** | | **Problems and possible explanations** | | **Possible solutions and conclusions** |
| --- | --- | --- | --- | --- |
| **Conceptual and planning phase** | | **General conditions**  **Rationale and field of research**  **Resources** | **New and complex project** between two departments (consisting of three disciplines) embedded in the Competence Network of Heart Failure, a subproject of the project “health related quality of life in heart failure patients”  - compulsory Basic Clinical Dataset (BCD) for all trials that could hardly be adapted  - conduct of the trial according to ICH/GCP new for study centre and investigators  **Ambitious, not sufficiently rooted study hypothesis**  - literature on the actual state of care is not robust, but stated a poor level of guideline implementation  - good evidence for intervention targets only for patients with LVSD, therefore is used as an inclusion criterion  - unknown prevalence of patients with LVSD in general practice  - no ICC values available for power calculation  - type of (educational) intervention (in groups) allowed only a synchronised design, limiting the recruitment phase to nine months  **Personal and financial limitations** (1 study nurse and ½ research assistant; no funding for echocardiography) | „**Tailoring to general practice**“  Targets of intervention and outcomes need **longer preparatory development**[1]  - conceptual phase needs involvement of a primary care researcher from the beginning  - representative description of patient population and actual state of care necessary  - allow adaptation (reduction) of basic clinical dataset  Grants need sufficient funding (one study nurse can be in charge for max 20 German general practices) |
| **Operational phase** | **Recruitment of investigators (GPs)** | **Field of research**  **Cost-benefit ratio from GPs perspective** | **Research fatigue of GPs and size of recruitment catchment area**  - Department of GP and HSR and a “teaching practice net” was in its infancy  - First generation of complex research projects was already underway (128 (56+39+33) practices)  - Until 2005 approx. 150 „teaching practices“  - A single mail out to the 750 GPs. GPs could respond with a pre-specified fax to show interest. With the help of the Principal investigator, we contacted selected GPs by telephone again from a pool of 281 GPs (of these 750) that had been at least once involved with the Department. 83 GPs showed interest, finally.  - Area of dissemination too close to the study centre or urban agglomeration (where patients are attached primarily in specialised care)  **26 of the interested GPs finally declined**  - unfavourable personal cost-benefit ratio: high time and effort for enrolment and documentation of patients (i.e. burden of trial investigator files)  - financial incentive: 50,- Euro per patient documentation; **additional incentive of 100,- per included patient was offered**  - 50% chance of allocation to Train the trainer course | **Extension of area of recruitment** resulting in longer distances for the participating GP and the study nurse  “Teaching practices” do usually not commit more to research  **Multicenter approach** with involvement of more study centres, ideally in research networks  **Preparation of GPs** to introduce them to their roles as investigator in a trial according to ICH/GCP  **Increase of incentives**  An “a posteriori” -TTT course was offered |
|  | **Recruitment of study participants** | **Case finding**  **Inclusion of patients in the trial** | **Case finding extensive, individualised or unfamiliar (20 GPs did not find eligible study participants)**  - Heterogeneous practice prevalence of patients with LVSD ( in health care system with free choice of care, e.g. patients are attached primarily in specialised care)  - Non-representative pilot test of case finding that showed to be feasible  - Multitude of different providers/systems of electronic medical records,  - GPs were not used to electronic case finding (support was offered repeatedly)  - Missing adequate coding (according to ICD) of heart failure or underlying diseases  - Study centre initially overestimated knowledge and cardiological understanding of GPs  **Barrier LVSD (37 GPs recruited 168 eligible patients)**  [**Knowledge**](http://dict.leo.org/ende?lp=ende&p=thMx..&search=knowledge) **gaps in management of LSVD among GPs**  - violation of inclusion criterion LVSD (40 patients)  - lack of understanding of the rationale for the inclusion criterion LVSD  - GPs diagnose and manage patients with CHF by clinical judgement  - lack of understanding how to interprete findings routine documentation (e.g. “Left Ventricular ejection fraction” , grade of “impairment of systolic function”)  - GPs did not implement the planned screening and diagnostic procedure or did not react timely if barriers occurred, e.g. did not ask timely for support of the study centre  **Non-availability of timely access to echocardiography in some cases**  **Missing documentation** of the assessment of the ventricular function in the routine documentation of echocardiographies or doctor’s reports  - refusal by cardiologists to document an additional case report form because of financial reasons | **Extension of area of recruitment** (see above)  **Additional project meetings** to **create awareness** and motivation, to work-up the enrolment schedule and to ask timely for support, if necessary (potential interference with the study outcome – observation bias!)  **These results reflect the need for the trial (i.e. an intervention) indirectly!**  **Funding for (e.g. mobile study) echocardiography** |
| **Clinical phase and analysis** | |  | no major problems |  |

Table S1: Problems and possible solutions according to each phase of the clinical trial

1. Campbell M, Fitzpatrick R, Haines A, Kinmonth AL, Sandercock P, Spiegelhalter D, Tyrer P: **Framework for design and evaluation of complex interventions to improve health.** *BMJ* 2000, **321:**694-696.
